# Photoinduced water oxidation by an organic ligand incorporated into the framework of a stable metal–organic framework†
†Electronic supplementary information (ESI) available: Additional structure figures, TGA curve, adsorption isotherms, UV-vis spectra, ESR spectra, GC spectra, PXRD patterns, and an additional cyclic voltammogram. CCDC 1003764. For ESI and crystallographic data in CIF or other electronic format see DOI: 10.1039/c5sc02679b


**DOI:** 10.1039/c5sc02679b

**Published:** 2015-11-11

**Authors:** Yun-Nan Gong, Ting Ouyang, Chun-Ting He, Tong-Bu Lu

**Affiliations:** a MOE Key Laboratory of Bioinorganic and Synthetic Chemistry , State Key Laboratory of Optoelectronic Materials and Technologies , School of Chemistry and Chemical Engineering , Sun Yat-Sen University , Guangzhou 510275 , China . Email: lutongbu@mail.sysu.edu.cn; b College of Chemistry and Chemical Engineering , Gannan Normal University , Ganzhou 341000 , China

## Abstract

A major challenge for the development of organic water oxidation catalysts is their low chemical stability and low catalytic efficiency. Herein, we first demonstrate that both the chemical stability and catalytic efficiency of an organic ligand for water oxidation can be improved by incorporating it into the framework of a stable MOF. This opens up a promising avenue for the development of stable and efficient organic water oxidation catalysts.

## Abstract 2

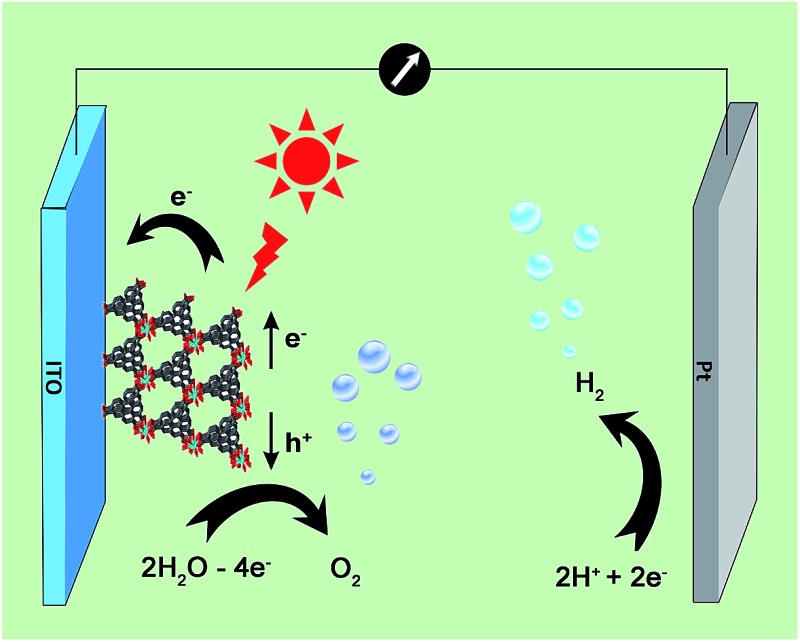
Both the chemical stability and the catalytic efficiency of an organic ligand (TTCA) can be enhanced during the photoinduced water splitting reaction by incorporating it into the framework of a stable MOF.

## Introduction

Photoinduced water splitting is one of the most promising ways for the direct conversion of solar energy into chemical fuels.^1^ Water splitting is a four-electron and four-proton transfer process, which oxides water to oxygen and reduces protons to hydrogen. To achieve the above two half-reactions, a good photoinduced water splitting catalyst capable of driving photoinduced electron transfer (PET) and converting solar energy into chemical energy is essential, in which the photogenerated electrons can reduce protons to hydrogen, and the subsequently photogenerated holes can oxidize water to oxygen. Because the water oxidation reaction requires a multielectron transfer process achieved at a very high redox potential, this half reaction is considered as the bottleneck for achieving water splitting,^2^ as the water oxidation catalysts (WOCs) are easily decomposed under the high redox potential required to oxidize water. Over the past decade, significant progress has been achieved in the development of molecular complexes^3^ and nanomaterials^4^ as WOCs in photocatalytic water oxidation systems. Incorporating Ir WOCs into the frameworks of Zr-based MOFs has also been reported.^5^ However, water oxidation has rarely been achieved by organic compounds, and, to our knowledge, only two cases have been reported so far,^6^ probably due to their tendency to undergo decomposition under the high oxidation potential required for water oxidation. Organic water oxidation catalysts are potentially advantageous due to their low manufacturing cost,^6*b*^ as well as the option to change their structures to optimize their catalytic water oxidation efficiency. However, increasing the chemical stability of organic compounds is a major challenge for the development of organic water oxidation catalysts.

The chemical stability of an organic compound may be enhanced if it is incorporated into the framework of a metal–organic framework (MOF), and this can be easily achieved by using an organic compound as a linker to react with metal ions to form a MOF. Incorporating an organic compound into the framework of a MOF has the following advantages: (1) the chemical stability of the organic compound can be enhanced; (2) the conjugation of the organic ligand can be extended to the framework of the MOF, so the HOMO–LUMO energy gap will become smaller, which is beneficial for a PET process; (3) the extended conjugation in the framework can prolong the electron–hole recombination time within the framework, which can enhance the catalytic efficiency of the organic WOC. Herein, triphenylene-2,6,10-tricarboxylic acid (H_3_TTCA)^7^ was chosen as a model compound to react with metal ions to form a MOF as it is a π-conjugated organic ligand that is capable of undergoing a PET process. The results demonstrate that both the chemical stability and catalytic efficiency of an organic ligand for water oxidation can be improved by incorporating it into the framework of a stable MOF.

## Results and discussion

The solvothermal reaction of H_3_TTCA with La(NO_3_)_3_·6H_2_O and hydrochloric acid in DMF at 120 °C for 24 h led to the formation of colorless block-shaped crystals of [La(TTCA)(H_2_O)]·DMF·H_2_O (**1**) (TTCA^3–^ = triphenylene-2,6,10-tricarboxylate, DMF = *N*,*N*-dimethyl formamide). **1** can be used as a catalyst for photocatalytic water splitting to form molecular oxygen and hydrogen. To our knowledge, this is the first report of catalytic water splitting by an organic compound incorporated into the framework of a MOF.

**Fig. 1:**
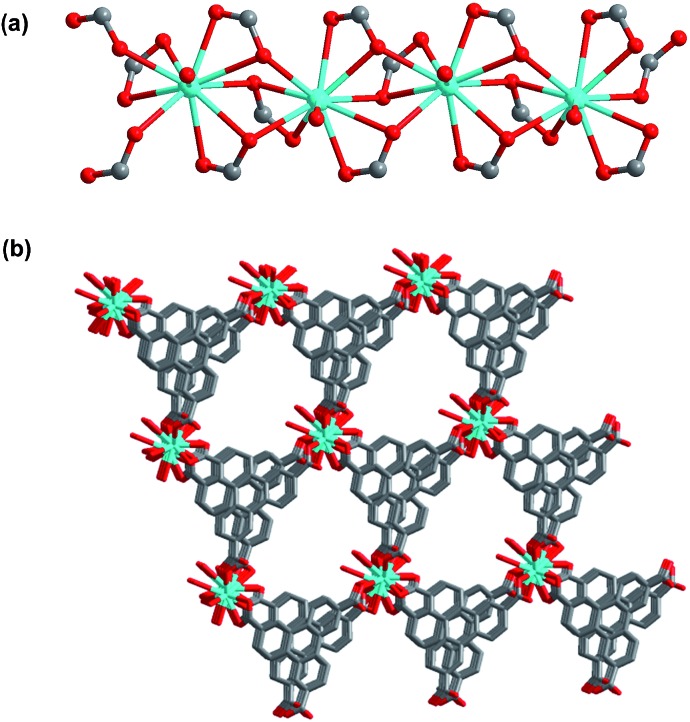
(a) View of the 1D La chain linked by μ_3_-CO_2_ groups in **1**; (b) the 3D framework of **1** with 1D channels.

The results of the single crystal X-ray structural analysis reveal that **1** crystallizes in the monoclinic space group *Cc*. In **1**, the La1 ion is ten-coordinated to nine oxygen atoms from six TTCA^3–^ ligands and one water molecule (Fig. S1†). Adjacent La ions are connected by μ_3_-CO_2_ groups to form a one-dimensional chain (Fig. 1a), with a La···La distance of 4.085 Å and a La···La···La angle of 166.772°. The 1D chains link to each other through TTCA^3–^ ligands, leading to a three-dimensional framework with one-dimensional channels along the *c* axis (Fig. 1b). The size of the channel is 6 × 8 Å. Each La ion, linking six TTCA^3–^ ligands, can be regarded as a six-connected node, and each TTCA^3–^ ligand, connecting six La ions, can also be considered as a six-connected node. Therefore, the overall structure can be simplified to a (6,6)-connected network with {4^9^·6^6^} topology (Fig. S2†). The pores of **1** are filled with disordered DMF and H_2_O molecules, and the calculated porosity (occupied by DMF and guest water molecules) by PLATON^8^ is 30%.

**Fig. 2:**
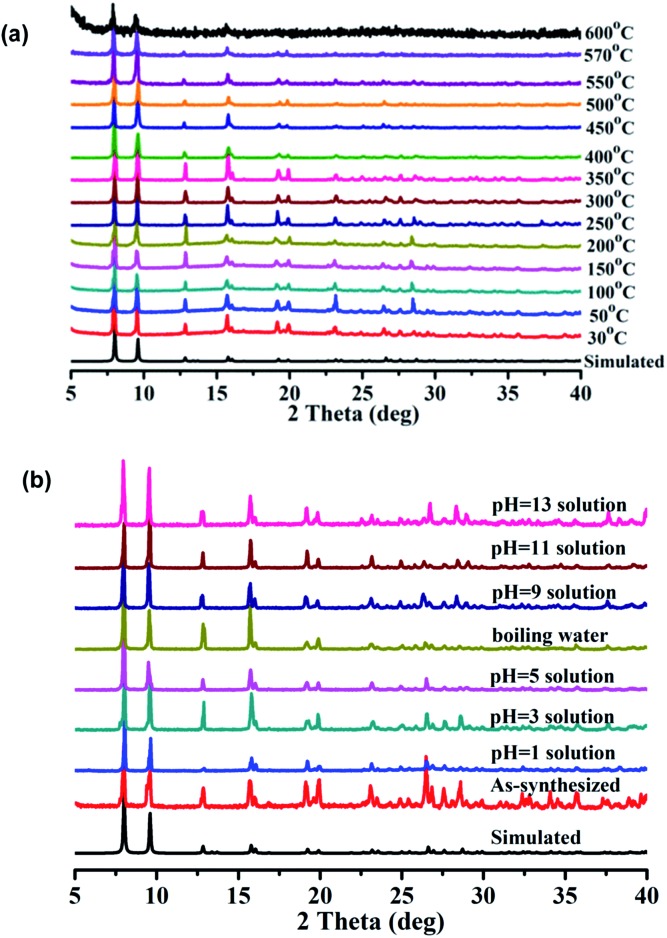
(a) The variable temperature PXRD patterns for **1**; (b) PXRD patterns for **1** soaked in boiling water and aqueous solutions with pH values ranging from 1 to 13 for 7 days.

The results of the thermogravimetric analysis (TGA) indicate that **1** readily lost DMF and H_2_O molecules in the temperature range of 30–320 °C, and desolvated **1** is stable up to 530 °C (Fig. S3†). The results of the variable temperature PXRD measurements demonstrate that the framework of **1** is stable up to 570 °C (Fig. 2a). In rare instances, the framework of a MOF can be stable above 500 °C.^9^ The high thermal stability of **1** may originate from the high thermal stability of the ligand, which can be stable up to 380 °C. The most striking feature is that **1** also exhibits a very high chemical stability. The results of the PXRD measurements demonstrate that **1** retains its framework not only in boiling water but also in aqueous solutions with pH values ranging from 1 to 13 for 7 days (Fig. 2b).

To further investigate the stability of **1**, N_2_ sorption of desolvated **1** was investigated after soaking **1** in boiling water and in aqueous solutions for 7 days with pH values of 1, 2, 12 and 13. The N_2_ adsorption results of those samples show almost complete maintenance of the porosity (Fig. S4 and Table S3†), demonstrating that the framework of **1** is, indeed, stable in both acidic and basic media, as well as in boiling water. In addition, almost no dissolved TTCA ligand was detected by UV-vis measurements of the filtrate after the above chemical treatments (Fig. S5 and Table S3†), further demonstrating that the framework of **1** was not decomposed. The high chemical stability of **1** can be attributed to the high coordination number of La(iii) and the formation of stable 1D La(iii) chains (Fig. 1a). Thus, the framework of **1** is resistant to attack from boiling water and even acidic and basic solutions. Considering the relatively weak La(iii)–oxygen coordination interactions, such unprecedented chemical stability is remarkable. To our knowledge, only one other such MOF displays a high chemical stability in the pH range of 2–12,^10^ where the high chemical stability was assigned to a combination of the high coordination number of La(iii), the triangular ligand units and the coordination type.^10^ A MOF that exhibits both a high thermal stability (up to 570 °C) and a high chemical stability (pH 1–13, and boiling water) has not been reported so far.

**Fig. 3:**
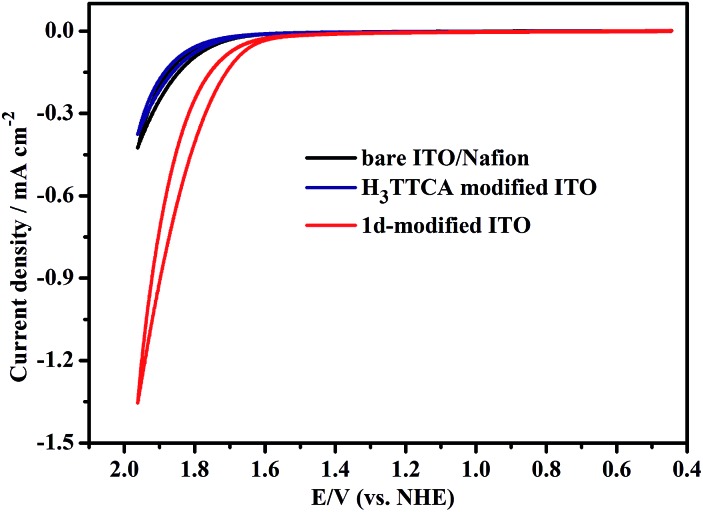
Cyclic voltammograms of the bare ITO/Nafion, H_3_TTCA-modified ITO and **1d**-modified ITO working electrodes in 0.2 M NaOAc/HOAc buffer solution (pH 5.0) irradiated with a high-pressure mercury lamp. Saturated calomel was used as the reference electrode (0.244 V *vs.* NHE), and Pt foil was sued as the counter electrode.

To see if the chemical stability and water catalytic efficiency of the organic TTCA ligand can be enhanced by incorporating it into the framework of **1**, its photoinduced water splitting properties were investigated. The H_3_TTCA-modified or **1d**-modified electrode used for the photocatalytic research was prepared by dropping PrOH/H_2_O/Nafion solution containing H_3_TTCA or dehydrated **1** (**1d**) onto an ITO glass slide, and allowing it to dry at room temperature.^11^ Cyclic voltammograms (CVs) of the **1d**-modified and bare ITO/Nafion (without catalyst) electrodes were obtained in 0.2 M NaOAc/HOAc buffer at pH 5.0 irradiated with a high pressure mercury lamp (450 W, *λ*_max_ = 365 nm). As shown in Fig. 3, the oxidation current density of the **1d**-modified electrode is 1.36 mA cm^–2^ at +1.96 V *versus* the normal hydrogen electrode (NHE), which is ∼3 times enhanced compared to that of the bare ITO/Nafion electrode (0.43 mA cm^–2^). This is consistent with photoinduced catalytic water oxidation. Furthermore, the current densities of the **1d**-modified electrode irradiated with visible light (*λ* > 420 nm) and the H_3_TTCA-modified electrode irradiated with a high pressure mercury lamp are 0.35 and 0.37 mA cm^–2^, respectively, at +1.96 V *versus* NHE (Fig. 3 and S6†). These values are close to that of 0.43 mA cm^–2^ for the bare ITO/Nafion electrode, demonstrating that the catalytic water oxidation of **1d** can only be induced by UV light rather than by visible light. This can be attributed to the fact that the maximum absorption of **1** appears at 363 nm (Fig. S7†). The organic ligand H_3_TTCA itself lacks catalytic water oxidation ability under the same conditions, as the maximum absorption of the ligand in NaOAc/HOAc buffer (pH 5.0) appears at 269 nm, and thus the electron can not be excited from the ground state to its excited state by a high pressure mercury lamp, and a PET process can not be achieved. From the above results it can be seen that incorporating the organic ligand TTCA into the framework of **1** can indeed reduce the HOMO–LUMO energy gap, which is beneficial for a PET process. This was confirmed by the results of density functional theory (DFT) calculations for the ligand H_3_TTCA and the complex of MOF **1**, in which the HOMO–LUMO energy gap decreased from 3.17 eV in the ligand to 2.72 eV in the MOF **1** (Fig. S8†).

**Fig. 4:**
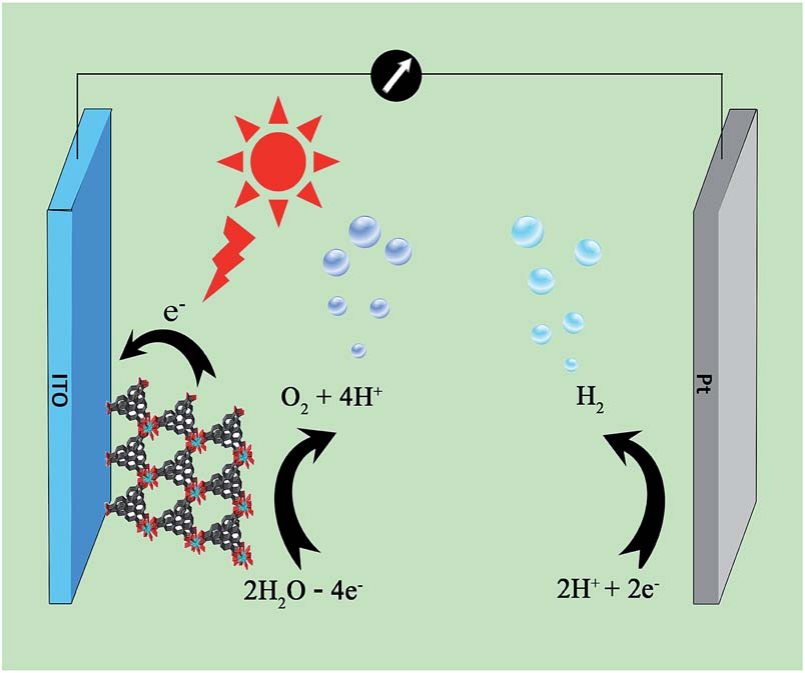
Schematic illustration of a PEC device consisting of a photoanode of **1d**-modified ITO, a Pt cathode, and 0.2 M NaOAc/HOAc buffer solution (pH 5.0) electrolyte, for UV light driven water splitting.

**Fig. 5:**
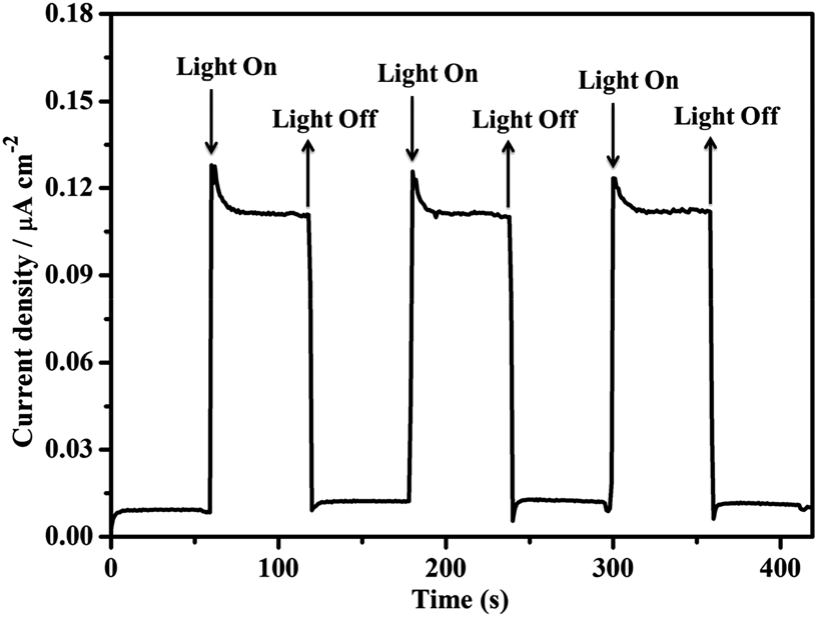
The transient short-circuit current responses to on–off cycles of illumination by a high-pressure mercury lamp in 0.2 M NaOAc/HOAc buffer solution (pH 5.0) in a PEC device with a 0.6 V (*vs.* NHE) bias.

A three-electrode photoelectrochemical cell (PEC) was built with **1d**-modified ITO as the photoanode, Pt foil as the cathode, saturated calomel as the reference electrode, and 0.2 M NaOAc/HOAc buffer at pH 5.0 as the electrolyte (Fig. 4). The transient short-circuit photocurrent was investigated with the on–off cycle's illumination of the **1d**-modified ITO photoanode in the above PEC when applying an external bias of +0.6 V (*vs.* NHE). As shown in Fig. 5, without illumination, it generates a transient dark current density nearly at zero. By contrast, upon illumination with a high-pressure mercury lamp, a dramatic enhancement of the photocurrent density was observed, and the photocurrent density slowly degrades to a steady state of 0.11 μA cm^–2^. When the UV light is switched off, the photocurrent density rapidly decays to nearly zero. The above transient short-circuit photocurrent responding to on–off cycles of illumination can be repeated several times (Fig. 5), and the above phenomenon can be attributed to a PET process. In this process, an electron is first excited from the ground state of TTCA in **1d** to its excited state by UV light, generating the ˙TTCA radical. This then transfers an electron to the Pt cathode, and subsequently reduces H^+^ to H_2_ and generates an oxidation state of TTCA^+^. Finally, the TTCA^+^ cation gains an electron from water to form TTCA, and water is oxidized to oxygen. A similar pathway has been previously reported,^6*a*^ in which the photoinduced water oxidation reaction can be achieved by a fluorescent active protonated cryptand containing an anthracene framework. The existence of the ˙TTCA radical can be monitored by ESR spectroscopy. As shown in Fig. S9†, upon illumination with a high-pressure mercury lamp, the solid of **1d** shows a signal at *g* = 2.0023, which can be attributed to the signal of the ˙TTCA organic radical.^12^ This signal disappeared upon stopping the illumination. In addition, no ESR signals were observed when the solid of **1d** was irradiated with visible light (*λ* > 420 nm). The above results demonstrate that the ˙TTCA radical can only be produced by UV light. To study the charge recombination behaviour of each UV light-on cycle, the transient decay time (*t*_ad_) was calculated according to the literature method^13^ (eqn (S1) and Fig. S10†). The transient time constant (*τ*) is defined as the decay time when ln *t*_D_ = –1.^13^ **1d** shows a *τ* value of up to 6.3 s (Fig. S11†), implying a slow charge recombination process for **1d**.^14^

**Fig. 6:**
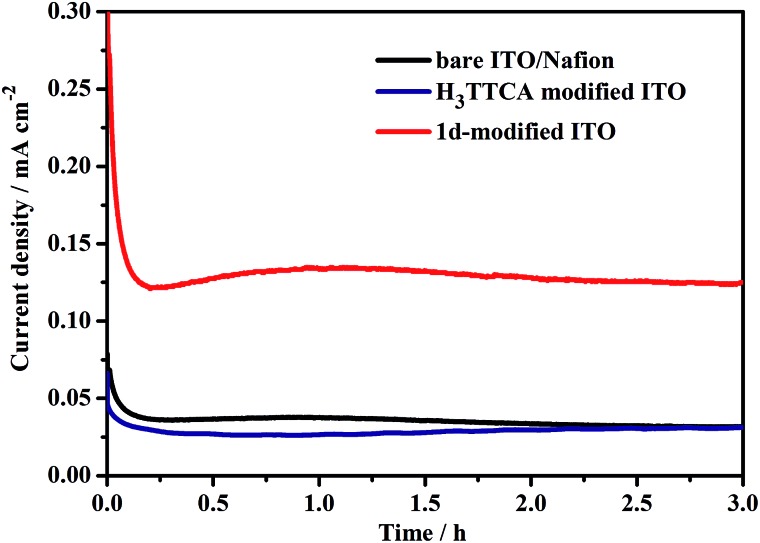
Three-electrode photocurrent measurements with a 1.96 V (*vs.* NHE) external bias for the bare ITO/Nafion, H_3_TTCA-modified ITO and **1d**-modified ITO photoanodes in 0.2 M NaOAc/HOAc buffer solution (pH 5.0) irradiated with a high-pressure mercury lamp.

To evaluate the catalytic efficiency, the UV light-driven (high pressure mercury lamp) water splitting was performed by this type of PEC device, applying a +1.96 V (*vs.* NHE) external bias in 0.2 M NaOAc/HOAc buffer at pH 5.0 under an Ar atmosphere. As shown in Fig. 6, a stable photocurrent density of 0.13 mA cm^–2^ was obtained. During this process, both O_2_ and H_2_ bubbles were released from the **1d**-modified photoanode and Pt cathode, respectively. After 3 h of UV light illumination, 3.81 μmol of O_2_ and 7.45 μmol of H_2_ were detected by gas chromatography (Fig. S12 and S13†). The ratio of H_2_/O_2_ was 1.96, demonstrating that the electrons for reducing H^+^ to H_2_ originate from the water oxidation rather than acetate oxidation. The O_2_ generation turnover number (TON) was calculated to be 55 with a turnover frequency (TOF) value of 18.3 h^–1^ based on the catalyst **1d** (per TTCA molecule). When the photocatalytic experiment was extended to 10 h, the photocurrent was slightly increased rather than decreased (Fig. S14†), and 13.4 μmol of O_2_ and 25.4 μmol of H_2_ were obtained, with TON and TOF values of 193 and 19.3 h^–1^, respectively, demonstrating the high stability of the photocatalytic system. In comparison with the TON value of 13 achieved by the first organic WOC for electrocatalytic water oxidation at a potential of 2.2 V *versus* NHE,^6*b*^ our results show significant progress for the development of efficient organic WOCs. By contrast, under the same conditions, the current density of the H_3_TTCA-modified photoanode (0.022 mA cm^–2^) is close to that of the bare ITO/Nafion photoanode without catalyst decoration (0.036 mA cm^–2^, Fig. 6), which is much smaller than that for the **1d**-modified photoanode (0.13 mA cm^–2^). In these experiments, only trace amounts of H_2_ and O_2_ were detected by gas chromatography after 3 h of irradiation (0.76 μmol of H_2_ and 0.22 μmol of O_2_ for the H_3_TTCA-modified photoanode, and 0.40 μmol of H_2_ and 0.14 μmol of O_2_ for the bare ITO/Nafion), demonstrating that the catalytic efficiency of the organic WOC (TTCA) was enhanced after incorporating it into the framework of a stable MOF. After the above 3 h water splitting experiments, the results of the PXRD measurements demonstrated that the framework of **1d** remains (Fig. S15†), and only 4% of dissolved La(iii) and 4% of dissolved TTCA were detected in solution by ICP-MS. This indicates that 4% of **1d** decomposed during the above water splitting experiments. On the other hand, 41% of the TTCA ligand decomposed under the same conditions (Fig. S16†), demonstrating that the chemical stability of the organic WOC can be dramatically enhanced during photoinduced water splitting after it has been incorporated into the framework of **1**.

## Conclusions

In summary, we first demonstrate here that both the chemical stability and the catalytic efficiency of an organic ligand (TTCA) can be enhanced during the photoinduced water splitting reaction once it is incorporated into the framework of a stable MOF (**1d**). The enhanced catalytic efficiency is due to the reduced HOMO–LUMO energy gap, which is beneficial for a PET process, as well as the slow charge recombination process after it is incorporated into the framework of a stable MOF. This approach opens up a promising avenue for the development of stable and efficient organic water oxidation catalysts. Even though the catalytic efficiency of TTCA is enhanced after it is incorporated into the framework of MOF **1d**, the external bias (1.96 V) is still very high, probably due to the poor conductivity of the MOF. In the next step, we will try to increase the conductivity of MOFs to see if the bias could be decreased.

## Experimental section

### Materials and instrumentation

All of the chemicals are commercially available and were used without further purification. UV-vis spectra were recorded on a Shimadzu UV-2501PC spectrophotometer. The ESR spectra were measured on a Bruker ER-420 spectrometer with a 100 kHz magnetic field in X band and an electronic field of 9655.448 MHz at room temperature. Elemental analysis was determined using an Elementar Vario EL elemental analyzer. The IR spectra were recorded in the 4000 to 400 cm^–1^ region using KBr pellets and a Bruker EQUINOX 55 spectrometer. The thermogravimetric analysis (TGA) was carried out on a Netzsch TG-209 Thermogravimetry Analyzer in a N_2_ atmosphere. The powder X-ray diffraction patterns were recorded on a D8 ADVANCE X-ray diffractometer. The N_2_ adsorption isotherms were measured with a BELSORP-max gas adsorption instrument. The electrochemical measurements were carried out using an electrochemical workstation CHI 620E. The electrolyte was 0.2 M NaOAc/HOAc buffer at pH 5.0 in water. Pt foil was used as the counter electrode in the three-electrode system. The reference electrode was a saturated calomel electrode (0.244 V *vs.* NHE) and the working electrodes were ITO slides. All potentials were reported *vs.* NHE. The amounts of H_2_ and O_2_ were determined using Agilent 7820 gas chromatography. The concentration of La(III) in the electrolyte after the water splitting experiment was measured by iCAP Qc inductively coupled plasma mass spectrometry (ICP-MS).

### Synthesis

#### [La(TTCA)(H_2_O)]·DMF·H_2_O (**1**)

A mixture of La(NO_3_)_3_·6H_2_O (0.043 g, 0.1 mmol), H_3_TTCA (0.018 g, 0.05 mmol), hydrochloric acid (1 drop) and DMF (8.0 mL) was heated at 120 °C for 24 h in a sealed Teflon-lined autoclave. The autoclave was cooled over a period of 18 h at a rate of 5 °C h^–1^. Colorless block-shaped crystals of **1** were collected by filtration. Yield: 60%. Anal. calcd for C_24_H_20_NO_9_La (**1**): C, 47.62; H, 3.33; N, 2.31%; found: C, 48.12; H, 3.45; N, 2.69%. IR (KBr, cm^–1^): 3403 (s), 1658 (vs), 1616 (s), 1588 (s), 1543 (s), 1496 (m), 1378 (s), 1294 (m), 1102 (m), 1031 (w), 896 (m), 868 (s), 792 (s), 698 (m).

### X-ray crystallography

The single-crystal data of **1** was collected on an Agilent Technologies Gemini A Ultra system, with Cu/Kα radiation (*λ* = 1.54178 Å). All empirical absorption corrections were applied using the SCALE3 ABSPACK program.^15^ The structure was solved by direct methods and refined by full-matrix least-squares analysis on *F* ^2^ using the SHELX97 program package. All non-hydrogen atoms were refined anisotropically. The electron density of the disordered DMF and H_2_O molecules in **1** were treated as a diffuse contribution using the program SQUEEZE.^16^ All calculations were performed using the SHELXTL system of computer programs.^17^ The crystallographic data for **1** are given in Table S1,† and the selected bond lengths and angles are given in Table S2.†

### Stability test for **1**

Experimental details for the stability test: pH = 1–6 HCl and pH = 8–13 NaOH aqueous solutions were prepared. Then, 150 mg of the sample was soaked in each of the above solutions and also in boiling water for 7 days. The sample was then filtered and dried for further characterization. Powder XRD studies show that all of these samples possess high crystallinity. The results of the UV-vis measurements indicate that almost no dissolved ligand was detected in the filtrate. The N_2_ uptakes for the samples after soaking in boiling water and in solutions with pH values of 1, 2, 12, 13 for 7 days were measured. In addition, the stability of **1** after the water splitting experiment was also checked by PXRD measurements.

### N_2_ sorption measurements

N_2_ adsorption isotherms for **1** before and after the treatments in the diverse solutions were measured with a BELSORP-max gas adsorption instrument. The cryogenic temperature of 77 K required for N_2_ sorption tests was controlled using a liquid nitrogen bath. The initial outgassing process for the sample was carried out under a high vacuum (less than 10^–6^ mbar) at 280 °C for 4 h. The desolvated sample and sample tube were weighed precisely and transferred to the analyzer.

### Computational details

The frontier orbitals were calculated using the Dmol^3^ procedure in the Materials Studio 5.0 package^18^ through spin-polarized density functional theory (DFT). The widely used generalized gradient approximation (GGA) with the Perdew–Burke–Ernzerhof (PBE) functional and the double numerical plus polarization (DNP) basis set as well as the DFT semi-core pseudopots (DSPP) were used. In order to reduce the cost, the calculations of MOF **1** were performed in the primitive cell but maintaining the crystal symmetry, and the orbital calculations were adopted gamma point only. Besides, all the calculations were just considered under vacuum conditions.

### Fabrication of the working electrode

Approximately 2 mg of desolvated **1** or H_3_TTCA ligand was added into 1 mL of the mixture H_2_O (650 μL)/PrOH (250 μL)/Nafion (100 μL), and sonicated for 30 min to disperse **1d** or H_3_TTCA. About 20 μL of the suspension was added onto an ITO glass slide (1 cm × 2 cm), and dried at room temperature to obtain the **1d**- or H_3_TTCA-modified working electrode. In addition, the bare ITO/Nafion working electrode without any sample was also prepared under the same conditions.

### Cyclic voltammetry

Cyclic voltammogram (CV) measurements were conducted in 0.2 M NaOAc/HOAc buffer at pH 5.0 under an air atmosphere, using bare ITO/Nafion, **1d**- and H_3_TTCA-modified ITO as working electrodes, which were irradiated with UV (high-pressure mercury lamp) or visible light. Saturated calomel was used as the reference electrode (0.244 V *vs.* NHE), and Pt foil was used as the counter electrode.

### Photoinduced water splitting

A **1d**-modified ITO or bare ITO/Nafion photoanode, a platinum foil cathode, and a saturated calomel reference electrode were placed in a gas-tight single-compartment cell filled with 25 mL of aqueous solution containing 0.2 M NaOAc/HOAc electrolyte at pH 5.0. The cell was sealed by a septa and the solution was degassed using high purity argon at room temperature for two hours prior to the experiment. The light-driven water splitting reaction was performed for 3 hours by irradiation with a high pressure mercury lamp, and by applying an external bias of 1.96 V (*vs.* NHE). The generated H_2_ and O_2_ in the headspace were quantified by gas chromatography with a thermal conductive detector with argon as the carrier gas.

## References

[cit1] Rüttinger W., Dismukes G. C. (1997). Chem. Rev..

[cit2] Li F., Zhang B. B., Li X. N., Jiang Y., Chen L., Li Y. Q., Sun L. C. (2011). Angew. Chem., Int. Ed..

[cit3] Xu Y. H., Fischer A., Duan L. L., Tong L. P., Gabrielsson E., Åkermark B., Sun L. C. (2010). Angew. Chem., Int. Ed..

[cit4] Kanan M. W., Nocera D. (2008). Science.

[cit5] Wang C., Xie Z. G., deKrafft K. E., Lin W. B. (2011). J. Am. Chem. Soc..

[cit6] Hao H. G., Zheng X. D., Lu T. B. (2010). Angew. Chem., Int. Ed..

[cit7] Gong Y. N., Meng M., Zhong D. C., Huang Y. L., Jiang L., Lu T. B. (2012). Chem. Commun..

[cit8] Spek A. L., PLATON 99: A Multipurpose Crystallographic Tool, Utrecht University, Utrecht, The Netherlands, 1999.

[cit9] Loiseau T., Serre C., Huguenard C., Fink G., Taulelle F., Henry M., Bataille T., Férey G. (2004). Chem.–Eur. J..

[cit10] Duan J. G., Higuchi M., Krishna R., Kiyonaga T., Tsutsumi Y., Sato Y., Kubota Y., Takata M., Kitagawa S. (2014). Chem. Sci..

[cit11] Gao M. R., Xu Y. F., Jiang J., Zheng Y. R., Yu S. H. (2012). J. Am. Chem. Soc..

[cit12] Mirzakulova E., Khatmullin R., Walpita J., Corrigan T., Vargas-Barbosa N. M., Vyas S., Oottikkal S., Manzer S. F., Hadad C. M., Glusac K. D. (2012). Nat. Chem..

[cit13] Tafalla D., Salvador P., Benito R. M. (1990). J. Electrochem. Soc..

[cit14] Sun Y. F., Sun Z. H., Gao S., Cheng H., Liu Q. H., Piao J. Y., Yao T., Wu C. Z., Hu S. L., Wei S. Q., Xie Y. (2012). Nat. Commun..

[cit15] Sheldrick G. M., SADABS, Program for Empirical Absorption Correction of Area Detector Data, University of Göttingen, Göttingen, 1996.

[cit16] van der Sluis P., Spek A. L. (1990). Acta Crystallogr., Sect. A: Found. Crystallogr..

[cit17] Sheldrick G. M., SHELXS 97, Program for Crystal Structure Refinement, University of Göttingen, Göttingen, 1997.

[cit18] Accelrys, Materials Studio Getting Started, release 5.0, Accelrys Software, Inc., San Diego, CA, 2009.

