# Supplementary material for: Photoinduced water oxidation by an organic ligand incorporated into the framework of a stable metal–organic framework
Source: Chem Sci. 2015 Nov 11;7(2):1070–5. doi: 10.1039/c5sc02679b (PMC5590092; doi:10.1039/c5sc02679b)
Supplement: Supplementary file 1 [file SC-007-C5SC02679B-s001.pdf]

Electronic Supplementary Material (ESI) for Chemical Science.
This journal is © The Royal Society of Chemistry 2015

Supporting Information

Photoinduced water oxidation by an organic ligand incorporated into the framework of a stable metal-organic framework

Yun-Nan Gong,[a,b] Ting Ouyang,[a] Chun-Ting He [a] and Tong-Bu Lu*[a]

[a] MOE Key Laboratory of Bioinorganic and Synthetic Chemistry, State Key Laboratory of Optoelectronic Materials and Technologies, School of Chemistry and Chemical Engineering, Sun Yat-Sen University, Guangzhou 510275 (China)

[b] College of Chemistry and Chemical Engineering, Gannan Normal University, Ganzhou 341000 (China)

Transient Time. The transient decay time was calculated according to the following formula:

$$t_D = \frac{I_t - I_{st}}{I_{in} - I_{st}} \quad \textbf{(S1)}$$

where I_{in} and I_t stand for the photocurrent densities at 0 s and t s, I_{st} represents the steady-state photocurrent density (Fig. S9).

Table S1. Crystal Data and Structure Refinements for **1**

	**1**
Formula	$C_{24}H_{20}NO_9La$
Fw.	605.32
Crystal system	monoclinic
Space group	*Cc*
a (Å)	18.4394(5)
b (Å)	13.7828(3)
c (Å)	8.1160(2)
α (°)	90
β (°)	92.319(2)
γ (°)	90
V (Å^3)	2060.96(9)
Z	4
D_c (g·cm^{-3})	1.657
Reflections/ Unique	5354 / 2453
R(int)	0.0421
GOF on F^2	1.077
$R_1[I \geq 2\sigma(I)]^a$	0.0373
$wR_2[I \geq 2\sigma(I)]^b$	0.0917

[a] $R_1 = \Sigma||F_o| - |F_c||/\Sigma|F_o|$. [b] $wR_2 = [\Sigma[w(F_o^2 - F_c^2)^2]/\Sigma w(F_o^2)^2]^{1/2}$, where $w = 1/[\sigma^2(F_o)^2 + (aP)^2 + bP]$ and $P = (F_o^2 + 2F_c^2)/3$.

Table S2. Selected Bond Distances (Å) and Angles (^{o}) for **1**.

**1**			
La(1)-O(1)	2.651(6)	La(1)-O(6)#2	2.635(5)
La(1)-O(1W)	2.578(6)	La(1)-O(3)#3	2.758(6)
La(1)-O(2)	2.593(5)	La(1)-O(4)#3	2.505(6)
La(1)-O(1)#1	2.606(6)	La(1)-O(3)#4	2.509(6)
La(1)-O(5)#2	2.673(6)	La(1)-O(6)#5	2.606(5)
O(1)-La(1)-O(1W)	128.32(18)	O(1W)-La(1)-O(6)#2	82.53(18)
O(1)#1-La(1)-O(3)#4	143.59(18)	O(3)#3-La(1)-O(3)#4	128.70(17)
O(1)-La(1)-O(2)	49.82(17)	O(1W)-La(1)-O(3)#3	64.66(17)
O(1)#1-La(1)-O(6)#5	145.20(17)	O(3)#3-La(1)-O(6)#5	115.50(17)
O(1)-La(1)-O(1)#1	102.01(18)	O(1W)-La(1)-O(4)#3	77.2(2)
O(5)#2-La(1)-O(6)#2	49.43(16)	O(3)#4-La(1)-O(4)#3	138.98(18)
O(1)-La(1)-O(5)#2	77.13(16)	O(1W)-La(1)-O(3)#4	70.36(19)
O(3)#3-La(1)-O(5)#2	108.74(16)	O(4)#3-La(1)-O(6)#5	79.62(18)
O(1)-La(1)-O(6)#2	126.54(17)	O(1W)-La(1)-O(6)#5	68.20(18)
O(4)#3-La(1)-O(5)#2	148.74(17)	O(3)#4-La(1)-O(6)#5	65.60(17)
O(1)-La(1)-O(3)#3	162.59(17)	O(1)#1-La(1)-O(2)	75.26(17)
O(3)#4-La(1)-O(5)#2	70.88(17)	O(2)-La(1)-O(5)#2	108.09(17)
O(1)-La(1)-O(4)#3	117.82(18)	O(2)-La(1)-O(6)#2	140.52(17)
O(5)#2-La(1)-O(6)#5	131.00(16)	O(2)-La(1)-O(3)#3	113.31(16)
O(1)-La(1)-O(3)#4	68.59(18)	O(2)-La(1)-O(4)#3	71.28(17)
O(3)#3-La(1)-O(6)#2	61.81(16)	O(2)-La(1)-O(3)#4	115.13(17)
O(1)-La(1)-O(6)#5	67.06(17)	O(2)-La(1)-O(6)#5	72.94(17)
O(4)#3-La(1)-O(6)#2	110.52(17)	O(1)#1-La(1)-O(5)#2	72.74(16)
O(1W)-La(1)-O(2)	133.35(19)	O(1)#1-La(1)-O(6)#2	67.29(17)
O(3)#4-La(1)-O(6)#2	89.68(17)	O(1)#1-La(1)-O(3)#3	65.63(17)
O(1)#1-La(1)-O(1W)	129.60(18)	O(1)#1-La(1)-O(4)#3	77.12(18)
O(6)#2-La(1)-O(6)#5	146.35(17)	O(3)#3-La(1)-O(4)#3	49.32(17)
O(1W)-La(1)-O(5)#2	116.60(19)		

Symmetry codes: #1: x, - y + 1, z - 1/2; #2: x - 1/2, y - 1/2, z - 1; #3: x - 1/2, y + 1/2, z - 1; #4: x - 1/2, -y + 1/2, z - 1/2; #5: x - 1/2, -y + 3/2, z - 1/2.

Table S3. N_2 Adsorption Results for the Samples of Desolvated **1** After Treating Under Different Conditions.

Treatment conditions [a]	N_2 uptake (STP $cm^3 \cdot g^{-1}$)	BET surface area ($m^2 \cdot g^{-1}$)	Total pore volume ($cm^3 \cdot g^{-1}$)	Decomposition [b]
As-synthesized	150	467	0.24	ND
Boiling water	148	462	0.24	ND
pH = 1	150	456	0.24	ND
pH = 2	153	468	0.24	ND
pH = 12	154	476	0.24	ND
pH = 13	154	462	0.25	0.07 %

[a] Boiling water, pH = 1, 2, 12, 13 correspond to the treatment conditions of soaking the samples of desolvated **1** in boiling water and in aqueous solutions for 7 days with pH values of 1, 2, 12 and 13, respectively.

[b] Calculated based on UV-vis standard curve of $TTCA^{3-}$ (Fig. S5). ND = No decomposition.

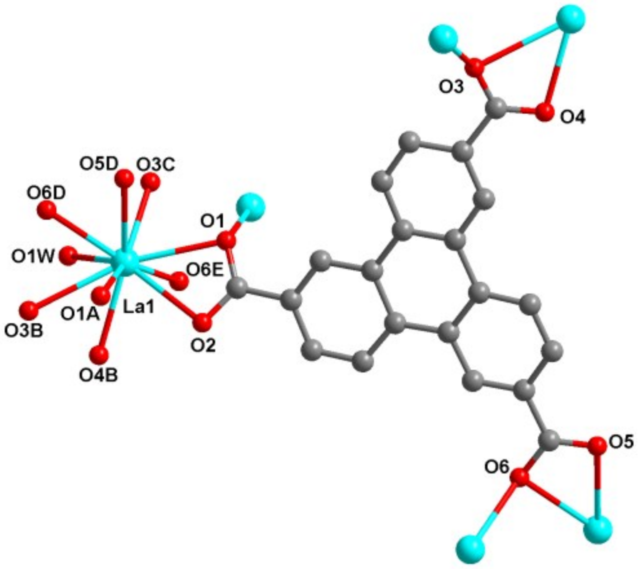

Fig. S1. The coordination environments of La(III) and $TTCA^{3-}$ anion in **1** (symmetry operations: A: x, - y + 1, z - 1/2; B: x - 1/2, y + 1/2, z - 1; C: x - 1/2, - y + 1/2, z - 1/2; D: x - 1/2, y - 1/2, z - 1; E: x - 1/2, - y + 3/2, z - 1/2).

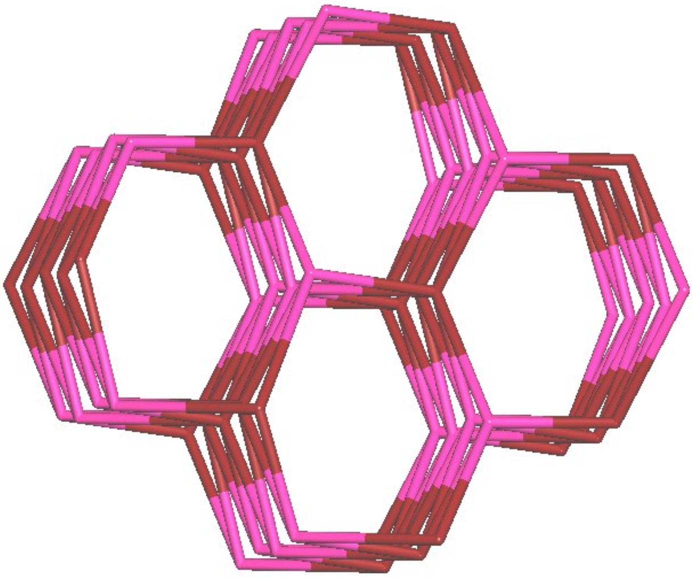

Fig. S2. 6,6-Connected 3D net derived from the structure of **1**. Pink: 6-connected nodes of TTCA ligands, brown: 6-connected nodes of La ions.

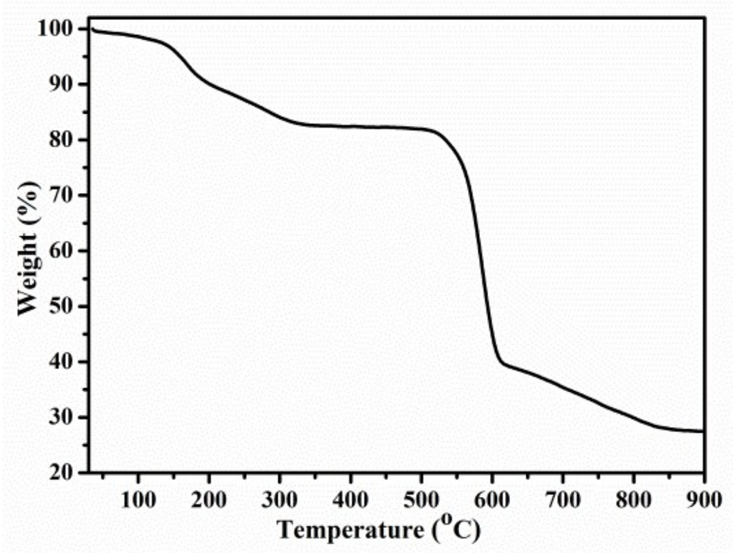

Fig. S3. TGA curve for **1**.

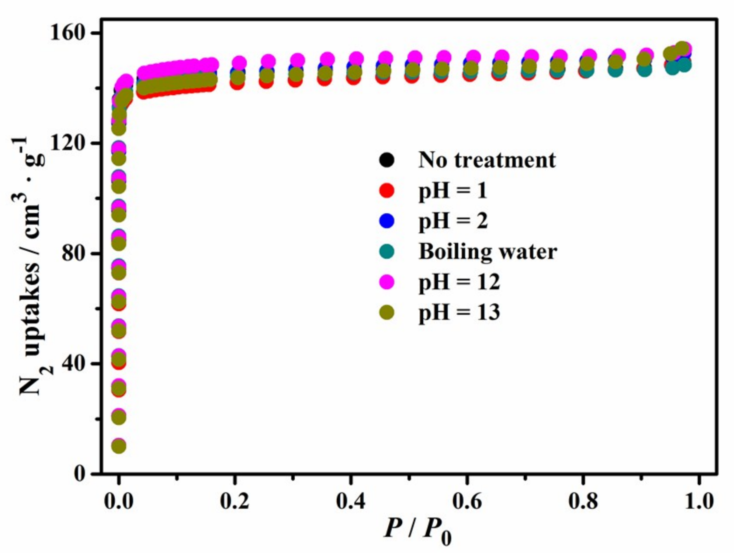

Fig. S4. N_2 sorption isotherms for **1d** before and after stability tests.

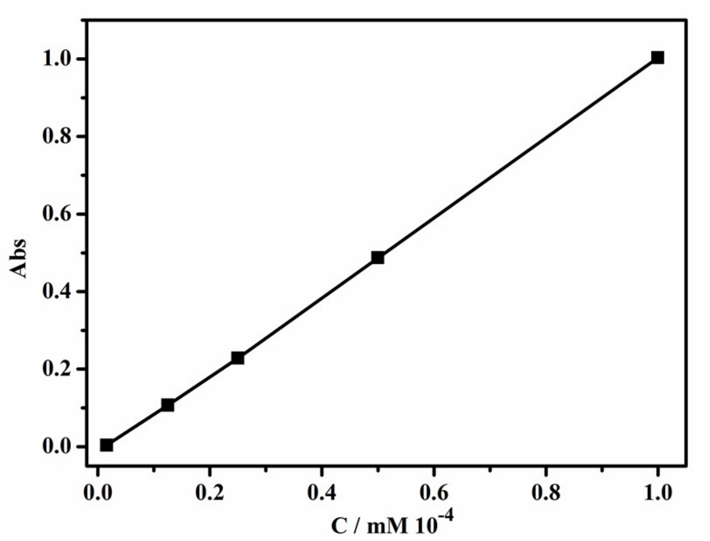

Fig. S5. The UV-vis standard curve of $TTCA^{3-}$ in aqueous solution.

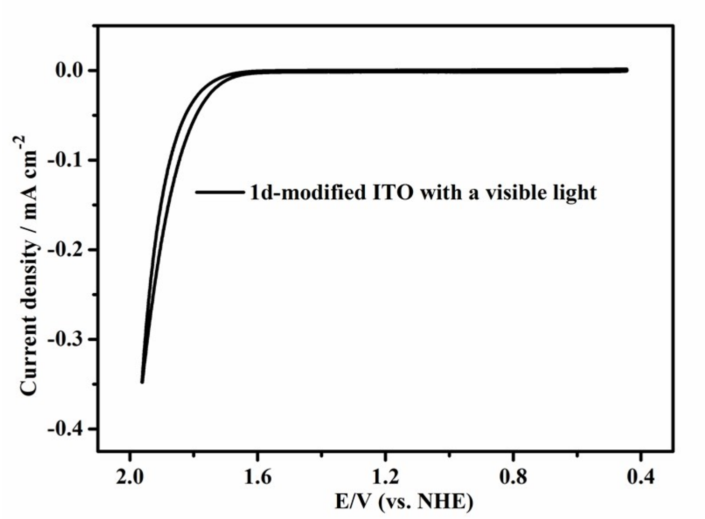

Fig. S6. Cyclic voltammogram of **1d**-modified electrode irradiated with a visible light (λ > 420 nm) in 0.2 M NaOAc/HOAc buffer at pH 5.0, saturated calomel as reference electrode (0.244 V vs. NHE), and Pt foil as counter electrode.

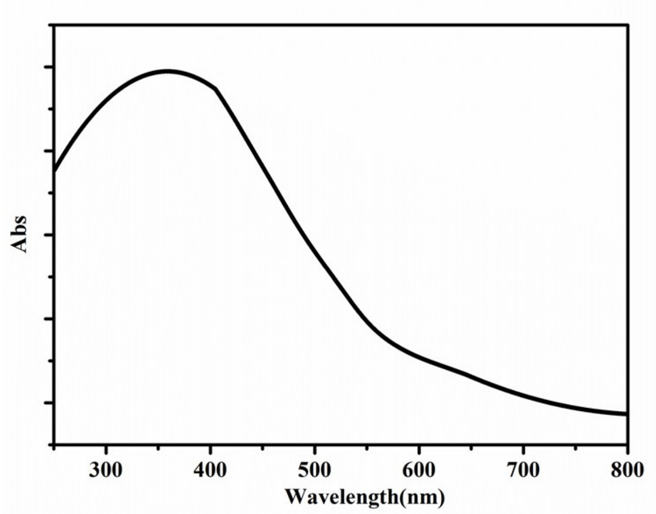

Fig. S7. The UV-vis diffuse-reflectance spectra of **1**.

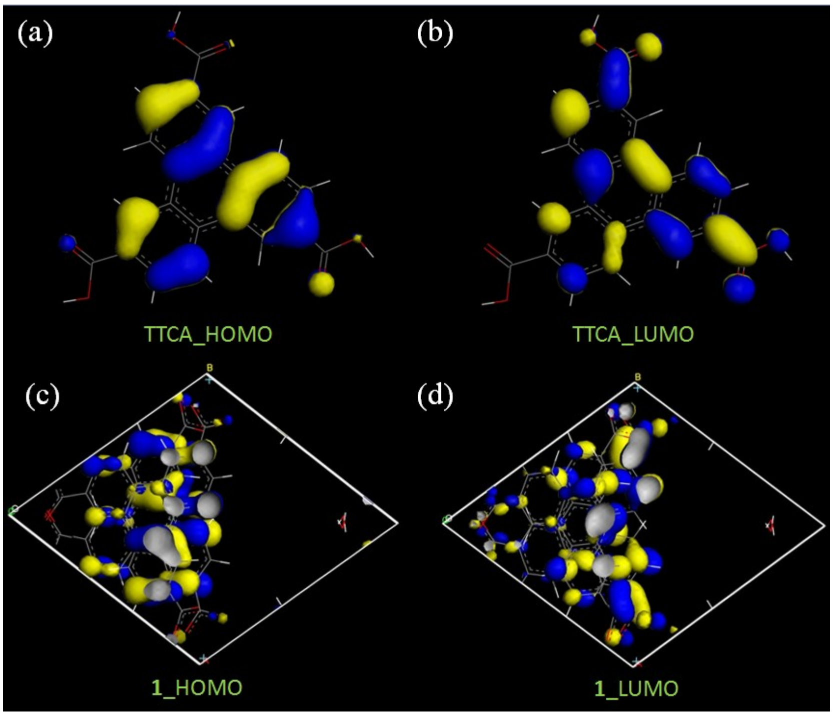

Fig. S8. HOMO and LUMO of the organic ligand TTCA (a, b) and the MOF **1** (c, d). (Note: Both HOMO and LUMO in the ligand and MOF **1** distribute on the organic components)

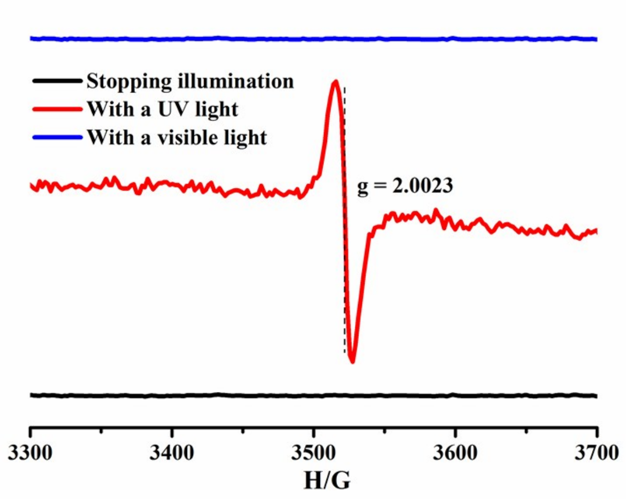

Fig. S9. The ESR spectra of the solid **1d** by irradiating with a high-pressure mercury lamp (red line) and a visible light (blue line), and stopping illumination (black line).

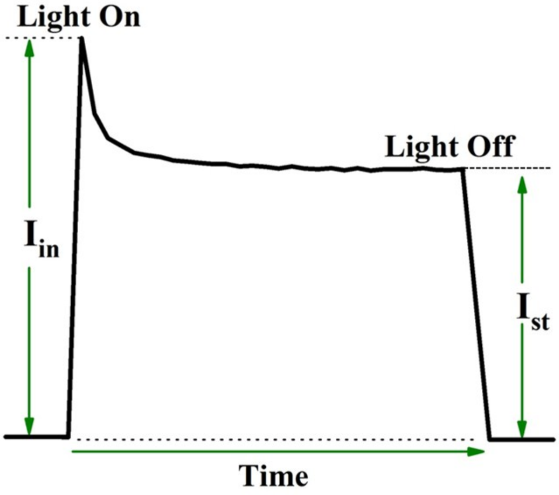

Fig. S10. A typical transient response photocurrent at a bias of 0.6 V vs. NHE.

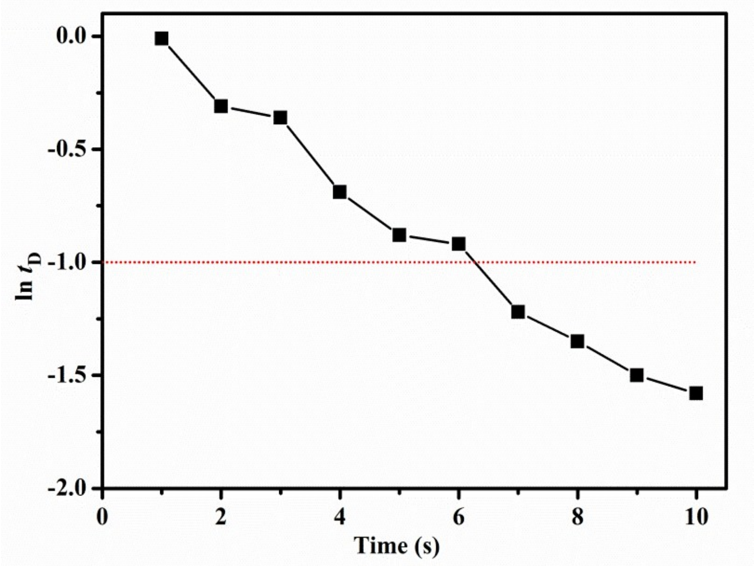

Fig. S11. The transient time constant of **1**.

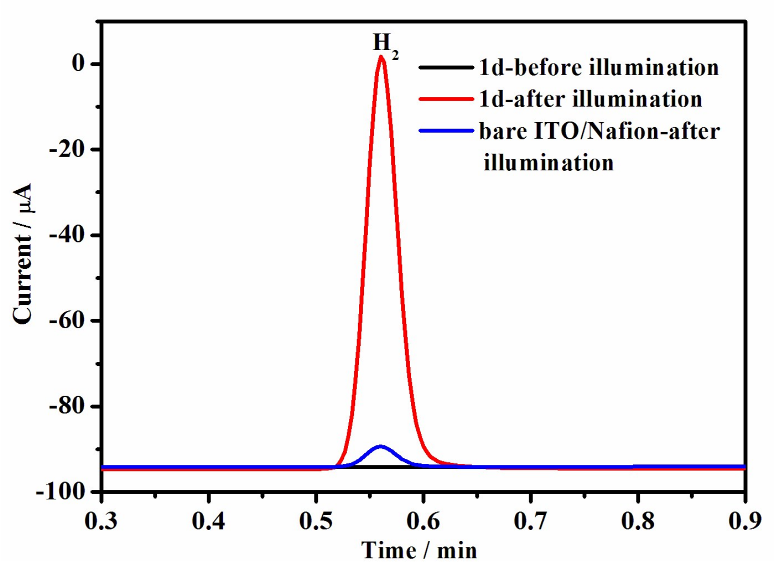

Fig. S12. GC data of H_2 for **1d**-modified electrode before (black line) and after (red line) light illumination, and for bare ITO/Nafion electrode after (blue line) light illumination for the PEC cell.

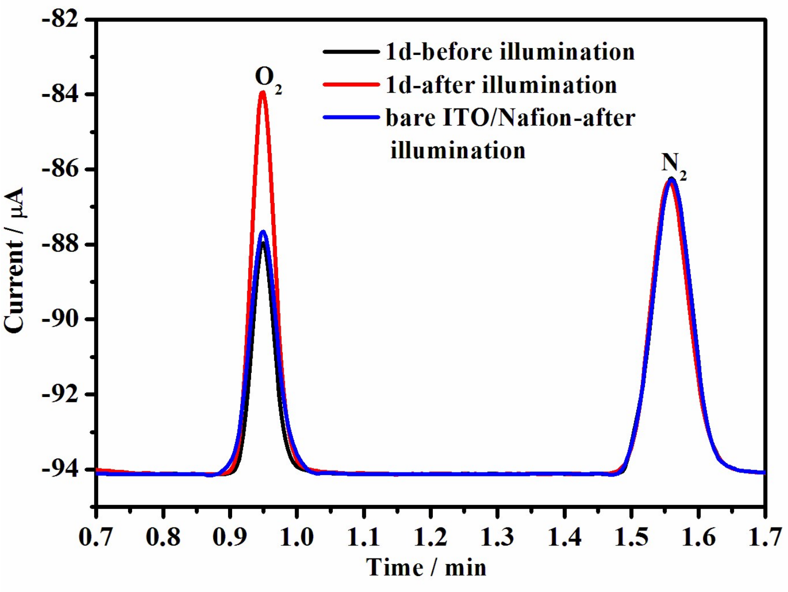

Fig. S13. GC data of O_2 and N_2 for **1d**-modified before (black line) and after (red line) light illumination, and for bare ITO/Nafion after (blue line) light illumination for the PEC cell. The intensity of O_2 (black line) comes from the residue of O_2 in the reaction system.

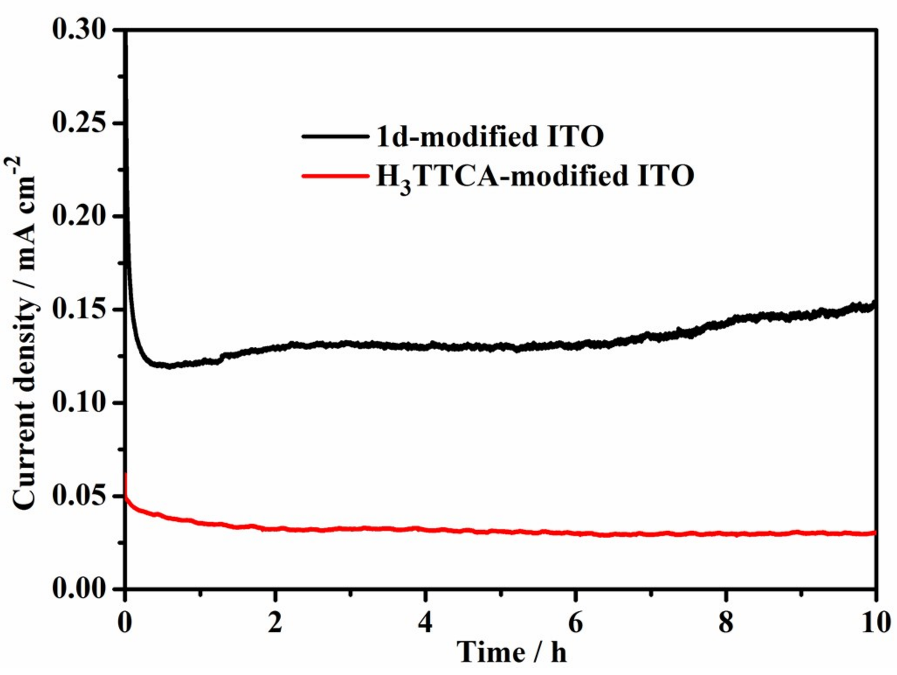

Fig. S14 Three-electrode photocurrent measurements with a 1.96 V (vs. NHE) external bias for H_3TTCA modified ITO and **1d**-modified ITO photoanodes in 0.2 M NaOAc/HOAc buffer solution (pH 5.0) for 10 h of UV light illumination.

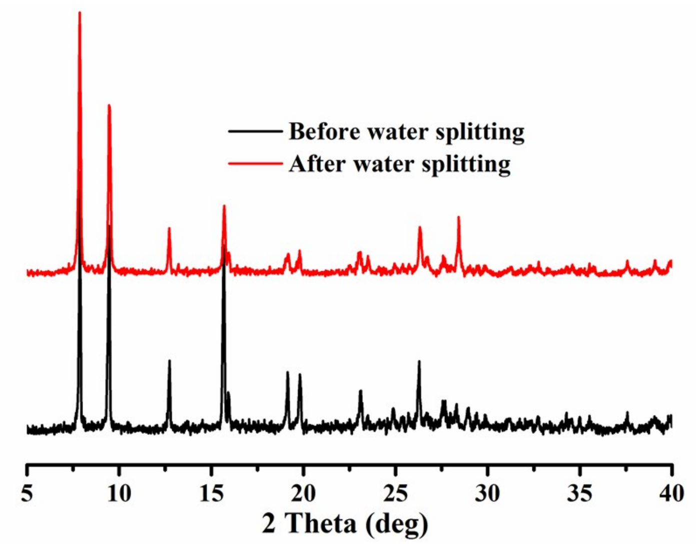

Fig. S15. The PXRD patterns of **1** before and after the water splitting experiment.

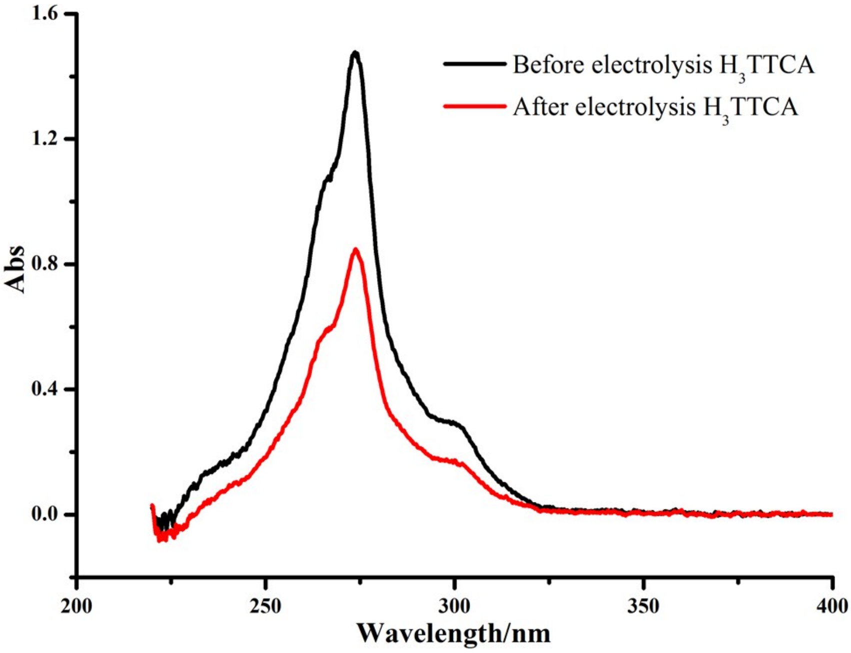

Fig. S16. The UV-vis spectra for the H_3TTCA-modified electrode before and after electrolysis in 0.2 M NaOAc/HOAc buffer solution (pH 5.0) irradiated with a high-pressure mercury lamp, the samples were obtained by dissolving the H_3TTCA in H_3TTCA-modified electrode after electrolysis (red line) and the same amount of H_3TTCA before electrolysis (black line) with 1.0 mL of 0.05 M NaOH, and 0.2 mL of

the above solution was dissloved in 4.8 mL of 0.2 M NaOAc/HOAc buffer solution (pH 5.0).
